# Dynamic Feature Elimination-Based Visual–Inertial Navigation Algorithm

**DOI:** 10.3390/s26010052

**Published:** 2025-12-20

**Authors:** Jiawei Yu, Hongde Dai, Juan Li, Xin Li, Xueying Liu

**Affiliations:** 1 Naval Aviation University, Yantai 264001, China; 2 School of Mathematics and Statistical Sciences, Ludong University, Yantai 264001, China; 3 Unit 92728 of the People’s Liberation Army, Shanghai 200436, China; 4 Unit 92742 of the People’s Liberation Army, Qinhuangdao 066000, China

**Keywords:** zero-velocity detection, gait cycle segmentation, adaptive threshold optimization, inertial navigation system

## Abstract

To address the problem of degraded positioning accuracy in traditional visual–inertial navigation systems (VINS) due to interference from moving objects in dynamic scenarios, this paper proposes an improved algorithm based on the VINS-Fusion framework, which resolves this issue through a synergistic combination of multi-scale feature optimization and real-time dynamic feature elimination. First, at the feature extraction front-end, the SuperPoint encoder structure is reconstructed. By integrating dual-branch multi-scale feature fusion and 1 × 1 convolutional channel compression, it simultaneously captures shallow texture details and deep semantic information, enhances the discriminative ability of static background features, and reduces mis-elimination near dynamic–static boundaries. Second, in the dynamic processing module, the ASORT (Adaptive Simple Online and Realtime Tracking) algorithm is designed. This algorithm combines an object detection network, adaptive Kalman filter-based trajectory prediction, and a Hungarian algorithm-based matching mechanism to identify moving objects in images in real time, filter out their associated dynamic feature points from the optimized feature point set, and ensure that only reliable static features are input to the backend optimization, thereby minimizing pose estimation errors caused by dynamic interference. Experiments on the KITTI dataset demonstrate that, compared with the original VINS-Fusion algorithm, the proposed method achieves an average improvement of approximately 14.8% in absolute trajectory accuracy, with an average single-frame processing time of 23.9 milliseconds. This validates that the proposed approach provides an efficient and robust solution for visual–inertial navigation in highly dynamic environments.

## 1. Introduction

In recent years, with the rapid development of computer vision technology, pure visual localization relies on visual data sources and is prone to interference from lighting changes and object occlusion. To address these issues, VIO (Visual–Inertial Odometry) technology has emerged [1,2,3]. By fusing multi-source data from visual cameras and IMU (Inertial Measurement Unit), VIO leverages the long-term robustness of vision [4] and the short-term high-precision characteristics of IMU [5]. This effectively compensates for the shortcomings of single sensors, making it a mainstream localization solution currently [6].

Classic algorithms based on the filtering framework, such as MSCKF [7] and ROVIO [8], achieve state estimation through recursive approximation and have advantages in computational efficiency. On the other hand, optimization-based algorithms like ORB-SLAM3 [9], VINS-Mono [10], and VINS-Fusion [11] adopt a tightly coupled front-end and back-end architecture. They use nonlinear optimization to improve global consistency and have become the mainstream direction of current research. Among them, VINS-Fusion, a representative work in the VINS series, fuses binocular vision and IMU data to achieve centimeter-level localization accuracy in static or low-dynamic scenarios. It is widely used in autonomous driving and robot navigation [12].

However, traditional VIO algorithms still face significant challenges in dynamic and complex scenarios. When the carrier is in traffic-dense urban environments, streets with heavy pedestrian flow, or indoor spaces with frequent object movements, visual sensors tend to capture interfering features from moving objects such as vehicles and pedestrians. These dynamic features are incorrectly used for pose estimation, leading to increased cumulative errors or even localization failure [13]. The core contradiction of this problem lies in the fact that traditional VIO assumes all feature points come from static backgrounds during feature processing, lacking an active mechanism to identify and eliminate dynamic interferences [14]. Therefore, enhancing adaptability to dynamic scenarios from the source of feature extraction while efficiently filtering dynamic interfering features has become the key to improving the performance of dynamic VIO.

Feature point extraction is a core part of the VIO front-end, and its performance directly affects the reliability of subsequent matching and optimization results [15]. Traditional feature extraction methods, such as SIFT [16] and SURF [17], rely on manually designed feature descriptors. Although they perform stably in static scenarios, they have high computational complexity and insufficient robustness to lighting and viewing angle changes [18]. Fast feature point algorithms represented by ORB [19] reduce computational complexity through binary descriptors but sacrifice the semantic expressiveness of features. This makes them prone to mismatching due to texture similarity in dynamic scenarios.

In recent years, deep learning-based feature extraction methods have automatically extracted features with high semantic value and invariance through self-supervised learning, significantly improving matching accuracy in complex scenarios [20]. Among these, SuperPoint [21], a representative work, adopts an encoder–decoder structure. It learns local key points and descriptors of images through self-supervised training, achieving better matching repeatability and localization accuracy than traditional methods in static scenarios [22]. However, the encoder design of SuperPoint still has limitations: the fusion of shallow and deep features is insufficient, making it difficult to effectively distinguish between static background and dynamic foreground feature points in dynamic scenarios. The surface texture of dynamic objects (such as moving vehicles) may contain rich local details, causing SuperPoint to misclassify them as reliable features, which are then incorrectly used for pose estimation.

To solve the interference problem of dynamic features, object tracking and multi-object motion estimation technologies have been introduced into the dynamic processing module of VIO. The SORT (Simple Online and Realtime Tracking) algorithm [23] realizes multi-object motion estimation through a “detection-tracking” framework. It first uses an object detection network to output bounding boxes of dynamic objects, then predicts object trajectories via Kalman filtering, and finally completes cross-frame matching based on the Hungarian algorithm. SORT has advantages in real-time performance and simplicity, but the Kalman filtering model with fixed noise covariance it adopts cannot adapt to the complexity of dynamic scenarios [24]. This leads to the accumulation of trajectory prediction errors, which in turn affects the elimination effect of dynamic features. Therefore, designing a more robust motion estimation model and combining feature-level and matching-level dynamic filtering mechanisms has become a key technology in the dynamic processing module of dynamic VIO.

To address the above challenges, this paper proposes an improved algorithm integrating multi-scale feature optimization and real-time dynamic point elimination based on the VINS-Fusion framework. In the front-end feature extraction stage, the encoder structure of SuperPoint is reconstructed to enhance its ability to distinguish static background features. In the dynamic processing stage, the ASORT (Adaptive Simple Online and Realtime Tracking) algorithm is designed. Combining adaptive extended Kalman filtering and multi-source information matching, it achieves accurate tracking of dynamic objects and efficient elimination of interfering features. Experiments show that this method significantly improves localization accuracy in dynamic scenarios while maintaining real-time performance, providing a new solution for visual–inertial navigation in complex environments.

## 2. Methods

**Figure 1:**
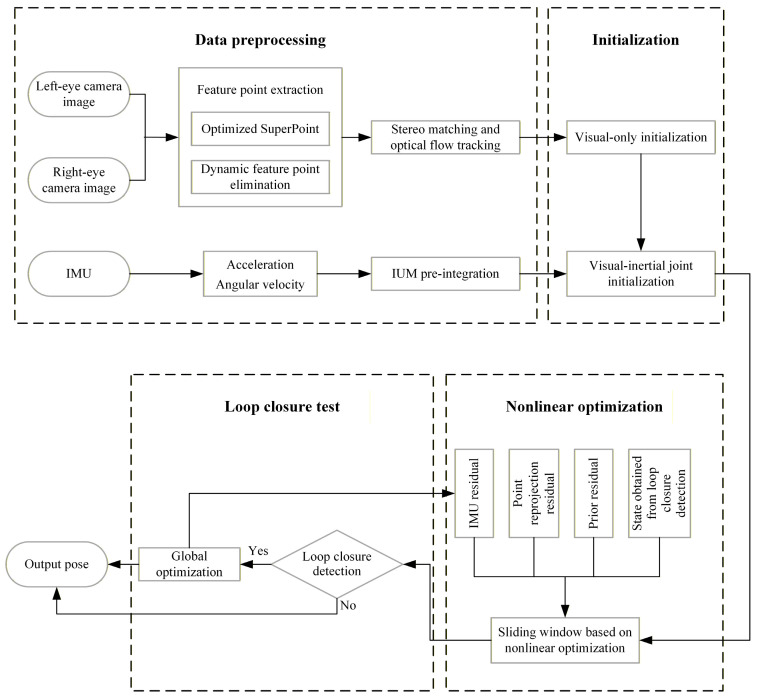
The complete structure of the algorithm mentioned in this article.

VINS-Fusion, as a classic stereo visual–inertial odometry algorithm, fuses data from stereo cameras and IMUs through a tight coupling approach. This framework mainly consists of three parts: front-end data processing, back-end nonlinear optimization, and loop closure detection, with its specific structure shown in Figure 1.

The front-end is responsible for extracting and tracking feature points from the stereo camera, while pre-integrating IMU data. The back-end performs pose estimation using nonlinear optimization methods, combined with the data provided by the front-end. The loop closure detection module uses a bag-of-words model to detect whether the carrier has returned to a previous position, so as to reduce cumulative errors. However, in dynamically complex scenarios, the traditional feature point extraction method in the front-end is easily disturbed by moving objects, resulting in a decrease in localization accuracy. To solve this problem, this paper proposes to introduce an optimized SuperPoint feature point extractor and a dynamic feature point elimination algorithm based on the VINS-Fusion framework, so as to improve the robustness and accuracy of the system in dynamic scenarios.

### 2.1. Optimization of SuperPoint Encoder with Multi-Scale Feature Fusion and Channel Compression

**Figure 2:**
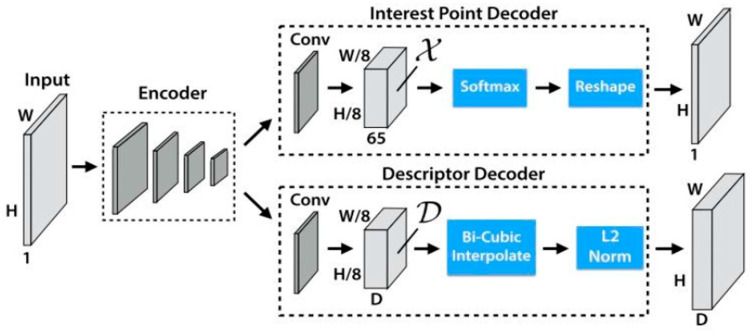
The Architecture Diagram of SuperPoint Algorithm.

SuperPoint is a feature point extraction and description network based on self-supervised learning, whose core implements feature point detection and description through an encoder–decoder structure. The algorithm uses a convolutional neural network to automatically learn key point positions and descriptors in images without the need for manually annotated data. Its self-supervised training strategy enables it to adapt to variations in illumination and viewing angles, exhibiting excellent matching accuracy and robustness in static scenarios. The algorithm structure is shown in Figure 2:

The algorithm accepts single-channel grayscale images or three-channel RGB (Red, Green, Blue) images subjected to weighted grayscale conversion, followed by normalization to eliminate the interference of illumination fluctuations on feature extraction. The shared encoder adopts a VGG (Visual Geometry Group)-style architecture: it takes preprocessed H×W×1 grayscale images as input, employs 8 convolutional layers, 3 max-pooling layers, and nonlinear activation layers, and outputs a feature map of size $\frac{1}{8}H\times \frac{1}{8}W\times 128$, whose primary function is to extract multi-scale features from the input image these features are then passed to two decoders, respectively, dedicated to key point detection and descriptor generation. The 128-dimensional feature map output by the encoder is simultaneously split into the key point decoding module and the descriptor decoding module, enabling parallel execution of key point detection and descriptor generation, and ultimately yielding two types of core results: first, a set of key points containing sub-pixel coordinates and detection confidence for each key point, where the confidence value can be used to further filter high-reliability key points; second, 128-dimensional normalized descriptor vectors in one-to-one correspondence with the key points, providing quantitative support for cross-frame feature matching.

**Figure 3:**
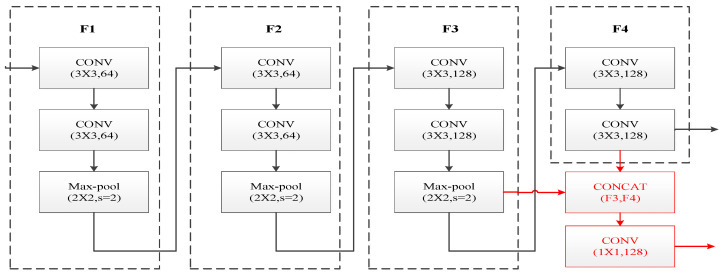
Encoder structure optimization diagram.

The encoder of SuperPoint suffers from insufficient fusion of shallow texture features and deep semantic features, which leads to easy confusion between static background and dynamic foreground features in dynamic scenarios. To address this issue, this paper reconstructs the encoder architecture through three-fold optimizations: dual-layer cascaded feature extraction, channel concatenation fusion, and 1 × 1 convolution dimensionality reduction, as shown in Figure 3. The red part indicates the improved sections of the original encoder proposed in this paper.

The encoder of SuperPoint adopts the classic VGG-style serial convolutional architecture, which was originally designed to extract deep semantic information of images through a layer-by-layer abstraction mechanism. However, this structure has inherent drawbacks in dynamic scenes: the high-resolution texture details captured by shallow convolutions are gradually diluted during the serial transmission in deep network layers. This semantic detail imbalance directly leads to the confusion of dynamic–static boundary features. When the algorithm attempts to eliminate dynamic feature points, static background features are prone to being misjudged as interference and wrongly removed, resulting in a reduction in the number of effective feature points and ultimately degrading the positioning performance of the VIO system in dynamic environments. Fundamentally, this issue arises because the single-path serial structure of traditional encoders cannot meet the requirements of multi-scale feature extraction. To address this fundamental contradiction, this paper innovatively reconstructs the encoder from an architectural perspective through three-layer optimization: dual-level cascaded feature extraction, channel concatenation and fusion, and 1 × 1 convolutional dimensionality reduction, as shown in Figure 3. The red parts in the figure indicate the improvements proposed in this paper to the original encoder.

To simultaneously capture local details and global semantics of the image, the improved encoder is designed with two parallel feature extraction branches, which output feature maps of the third layer ($F_{3}$) and the fourth layer ($F_{4}$), respectively, forming a multi-scale information foundation. The third-layer branch focuses on high-frequency detailed information such as local textures and edges of the image. After the input image of size H×W is processed by the first two convolutional layers, the feature map $F_{3}$ is output, where each spatial position (i,j) corresponds to a 128-dimensional feature vector, with the expression as follows:

(1) $$F_{3}=\left\{f_{3}^{\left(i,j\right)}\in R^{128}\left|1\leq i\leq \frac{H}{8}\right.,1\leq j\leq \frac{W}{8}\right\},$$

where $f_{3}^{\left(i,j\right)}$ denotes the feature representation at position (i,j).

The fourth-layer branch captures global semantic information of the image by increasing the receptive field, providing a semantic basis for distinguishing dynamic foreground regions from static background regions. Its structure is consistent with that of the third layer, and the expression of the output feature map $F_{4}$ is as follows:

(2) $$F_{4}=\left\{f_{4}^{\left(i,j\right)}\in R^{128}\left|1\leq i\leq \frac{H}{8}\right.,1\leq j\leq \frac{W}{8}\right\},$$

where $f_{4}^{\left(i,j\right)}$ denotes the feature representation at position (i,j).

Either $F_{3}$ or $F_{4}$ alone has information one-sidedness: using only $F_{3}$ tends to misjudge the local texture of dynamic objects as static features, while using only $F_{4}$ will lose the fine position information of static features. To address this, through channel concatenation, the 128-dimensional vector of $F_{3}$ and the 128-dimensional vector of $F_{4}$ are fused in the channel dimension to generate a 256-dimensional fused feature vector, achieving complementarity between details and semantics:

(3) $$F^{\left(i,j\right)}=concat\left(f_{3}^{\left(i,j\right)},f_{4}^{\left(i,j\right)}\right)\in R^{256}$$

The overall fused feature map is expressed as:

(4) $$F=\left\{F^{\left(i,j\right)}\in R^{256}\left|1\leq i\leq \frac{H}{8}\right.,1\leq j\leq \frac{W}{8}\right\}$$

Although the fused F has improved feature expression capability, its 256-dimensional channels will lead to a surge in subsequent computational load, which is inconsistent with real-time navigation requirements. To address this, 1 × 1 convolution is used for channel dimensionality reduction, outputting a feature vector ${\tilde{F}}^{\left(i,j\right)}\in R^{128}$ for each position (i,j):

(5) $${\tilde{F}}^{\left(i,j\right)}=\sigma \left(W*F^{\left(i,j\right)}+b\right),$$

where $W\in R^{1\times 1\times 256\times 128}$ represents the convolution kernel weight, $b\in R^{128}$ is the bias, σ(x)=max(0,x) denotes the ReLU activation function, and ∗ stands for convolution.

When expanded by components, the output of the k-th channel is:

(6) $${\tilde{F}}_{k}^{\left(i,j\right)}=\sigma \left(\sum_{256}^{c=1}W_{1,1,c,k}\cdot F_{c}^{\left(i,j\right)}+b_{k}\right),$$

where $W_{1,1,c,k}$ is the weight parameter of the convolution kernel from channel c to k. The compressed feature map is finally expressed as:

(7) $$\tilde{F}=\left\{{\tilde{F}}^{\left(i,j\right)}\in R^{128}\left|1\leq i\leq \frac{H}{8}\right.,1\leq j\leq \frac{W}{8}\right\}$$

This method achieves collaborative optimization of multi-scale feature extraction and computational efficiency by reconstructing the SuperPoint encoder architecture. The improved encoder adopts a parallel branch design to simultaneously extract shallow texture features and deep structural features. This multi-scale feature fusion mechanism significantly enhances the algorithm’s ability to capture stable feature points in static backgrounds, while suppressing interference from dynamic objects through the semantic feature branch. The improvement effect of this method on localization accuracy will be further verified through subsequent ablation experiments in this paper.

In terms of computational efficiency optimization, the introduced 1 × 1 convolution channel compression technology reduces network parameters and computational load by reconstructing feature dimensions while maintaining the expressive capability of feature maps. This optimization strategy improves the feature extraction speed of the algorithm and reduces computational complexity, providing an efficient solution for real-time localization applications.

### 2.2. Dynamic Feature Point Elimination Based on Adaptive Kalman Filtering

**Figure 4:**
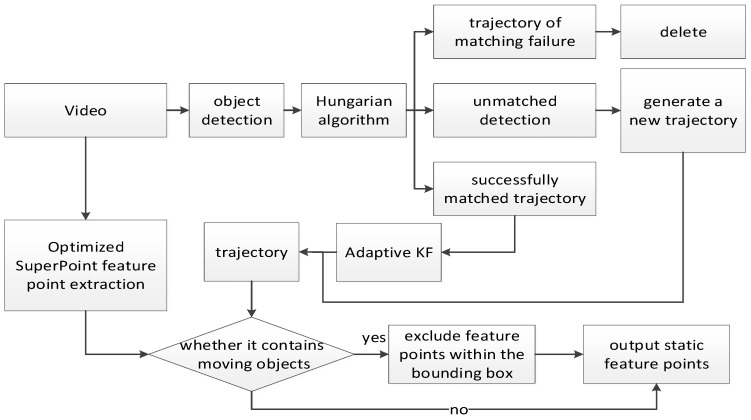
Dynamic feature point rejection flowchart.

The traditional SORT algorithm achieves multi-target tracking through target detection, Kalman filter prediction, and Hungarian matching, but it has two limitations in dynamic scenarios: first, it uses a standard Kalman filter, which can only handle linear systems and cannot adapt to the nonlinear motion of dynamic objects; second, it employs a fixed noise covariance model, making it difficult to cope with disturbances such as sudden changes in illumination and target occlusion, resulting in tracking drift. To address these issues, this paper proposes the ASORT algorithm, which adapts to nonlinear motion through KF, enhances robustness via adaptive noise adjustment, efficiently completes target detection and tracking, and combines with global feature points to achieve accurate filtering of dynamic feature points. The complete process of dynamic feature point elimination is shown in Figure 4.

The traditional SORT algorithm achieves multi-target tracking through object detection, Kalman filter prediction, and Hungarian matching, but it has two limitations in dynamic scenarios: first, it adopts the standard Kalman filter, which can only handle linear systems and cannot adapt to the nonlinear motion of dynamic objects; second, it uses a fixed noise covariance model, making it difficult to cope with disturbances such as sudden illumination changes and target occlusion, leading to tracking drift. To address these issues, this paper proposes the ASORT algorithm—as a front-end preprocessing module of the VIO system, its core function is to enhance robustness through adaptive observation noise adjustment, efficiently complete dynamic target detection and tracking, and realize accurate identification and elimination of dynamic feature points by combining global feature points before the feature points are input into the back-end optimization. The complete process of dynamic feature point elimination is illustrated in Figure 4:

#### 2.2.1. ASORT Algorithm

This paper adopts the YOLOv8-n lightweight target detection network, which has a small number of model parameters and fast inference speed, making it suitable for embedded devices and low-power scenarios. The network reduces computational load through depth-wise separable convolution while retaining the ability to detect small targets. The output result includes the observation vector $Z_{k}$ of the target at time k, which is a 4-dimensional vector directly output by the target detection network, corresponding to the parameters of the target bounding box:

(8) $$Z_{k}={\left[x_{c}^{o}(k),y_{c}^{o}(k),w^{o}(k),h^{o}(k)\right]}^{T},$$

where $x_{c}^{o}(k)$, $y_{c}^{o}(k)$, $w^{o}(k)$ and $h^{o}(k)$ correspond to the observed center coordinates, width, and height of the bounding box, respectively. In addition, it includes detection confidence, which is used to measure the reliability of the detection results.

First, state space modeling is performed, and the state vector $X_{k}$ of the target at time k is defined as:

(9) $$X_{k}={\left[x_{c}(k),y_{c}(k),w(k),h(k),v_{x_{c}}(k),v_{y_{c}}(k),v_{w}(k),v_{h}(k),a_{x_{c}}(k),a_{y_{c}}(k)\right]}^{T},$$

where $x_{c}(k)$ and $y_{c}(k)$ represent the horizontal and vertical coordinates of the center of the target bounding box, respectively; w(k) and h(k) denote the width and height of the target bounding box, respectively; $v_{x_{c}}(k)$ and $v_{y_{c}}(k)$ stand for the velocity components of the center point of the target bounding box in the x and y directions, respectively; $v_{w}(k)$ and $v_{h}(k)$ indicate the rate of change in the width and height of the target bounding box, respectively; and $a_{x_{c}}(k)$ and $a_{y_{c}}(k)$ represent the acceleration components of the center point of the target bounding box in the x and y directions, respectively.

The dynamic behavior of the target can be expressed as follows:

(10) $$X_{k}=f(X_{k-1})+W_{k},$$

where a is the process noise, assumed to be zero-mean Gaussian white noise with $W_{k}$ covariance matrix of Q. The process noise covariance matrix Q is set as a constant diagonal matrix based on the constant velocity model assumption, where the diagonal elements are configured according to the maximum acceleration of the target.

The state transition function is designed as follows:

(11) $$f(X_{k-1})=\left[\begin{array}{l} x_{c}(k-1)+v_{x_{c}}(k-1)\Delta t+\frac{1}{2}a_{x_{c}}(k-1)\Delta t^{2}\\ y_{c}(k-1)+v_{y_{c}}(k-1)\Delta t+\frac{1}{2}a_{y_{c}}(k-1)\Delta t^{2}\\ w(k-1)+v_{w}(k-1)\Delta t\\ h(k-1)+v_{h}(k-1)\Delta t\\ v_{x_{c}}(k-1)+a_{x_{c}}(k-1)\Delta t\\ v_{y_{c}}(k-1)+a_{y_{c}}(k-1)\Delta t\\ v_{w}(k-1)\\ v_{h}(k-1)\\ a_{x_{c}}(k-1)\\ a_{y_{c}}(k-1) \end{array}\right],$$

where Δt represents the inter-frame time interval.

The observation vector $Z_{k}$ of the system at time k and the state vector $X_{k}$ are related by the nonlinear observation function h(⋅):

(12) $$Z_{k}=h(X_{k})+V_{k},$$

where $V_{k}$ is the observation noise, assumed to be zero-mean Gaussian white noise with a covariance matrix of R. The initial observation noise covariance matrix R is set to a fixed value. This value is preset according to the characteristics of the dataset during algorithm initialization, which is consistent with the processing method of the classic SORT algorithm.

Based on the state estimation ${\hat{X}}_{k-1}$ at time k−1 and its estimation error covariance matrix ${\hat{P}}_{k-1}$, the state prior ${\hat{X}}_{k}^{-}$ and covariance prior ${\hat{P}}_{k}^{-}$ at time k are predicted.

(13) $${\hat{X}}_{k}^{-}=f({\hat{X}}_{k-1})$$

(14) $${\hat{P}}_{k}^{-}=F_{k}{\hat{P}}_{k-1}F_{k}^{T}+Q$$

The state transition matrix $F_{k}$ is expressed as follows:

(15) $$F_{k}=\left[\begin{array}{cccccccccc} 1 & 0 & 0 & 0 & \Delta t & 0 & 0 & 0 & \frac{1}{2}\Delta t^{2} & 0\\ 0 & 1 & 0 & 0 & 0 & \Delta t & 0 & 0 & 0 & \frac{1}{2}\Delta t^{2}\\ 0 & 0 & 1 & 0 & 0 & 0 & \Delta t & 0 & 0 & 0\\ 0 & 0 & 0 & 1 & 0 & 0 & 0 & \Delta t & 0 & 0\\ 0 & 0 & 0 & 0 & 1 & 0 & 0 & 0 & \Delta t & 0\\ 0 & 0 & 0 & 0 & 0 & 1 & 0 & 0 & 0 & \Delta t\\ 0 & 0 & 0 & 0 & 0 & 0 & 1 & 0 & 0 & 0\\ 0 & 0 & 0 & 0 & 0 & 0 & 0 & 1 & 0 & 0\\ 0 & 0 & 0 & 0 & 0 & 0 & 0 & 0 & 1 & 0\\ 0 & 0 & 0 & 0 & 0 & 0 & 0 & 0 & 0 & 1 \end{array}\right]$$

After obtaining the observation value $Z_{k}$ at time k, the state update is performed.

Calculate the Kalman gain:

(16) $$K_{k}={\hat{P}}_{k}^{-}H_{k}^{T}{\left(H_{k}{\hat{P}}_{k}^{-}H_{k}^{T}+R\right)}^{-1},$$

where $H_{k}$ is the state transition matrix of the observation function h(⋅) at point ${\hat{X}}_{k}^{-}$:

(17) $$H_{k}=\left[\begin{array}{cc} I_{4} & 0_{4\times 6} \end{array}\right]$$

Update the state estimation:

(18) $${\hat{X}}_{k}={\hat{X}}_{k}^{-}+K_{k}(Z_{k}-h({\hat{X}}_{k}^{-}))$$

Update the estimation error covariance matrix:

(19) $${\hat{P}}_{k}=(I-K_{k}H_{k}){\hat{P}}_{k}^{-}$$

This paper proposes a mechanism for adaptively adjusting the observation noise covariance, whose core idea is to dynamically adjust the effective observation noise level according to the confidence of the current observation:

(20) $$\hat{R}=(\frac{1}{2}+c_{k})R,$$

where $\hat{R}$ is the adjusted effective observation noise covariance matrix at time k, and $c_{k}\in \left[\frac{1}{2},1\right)$ is the adaptive factor for the current observation. The adaptive factor $c_{k}$ comprehensively considers the target motion state and lighting conditions:

(21) $$c_{k}=\sigma (\beta v+\delta \cdot \exp(-l\frac{\left|\lambda -\lambda_{0}\right|}{\lambda_{0}})),$$

where $\sigma (x)=\frac{1}{1+e^{-x}}$ and v are velocities, β is the velocity weight factor, λ is the current illumination value, $\lambda_{0}$ is the ideal illumination value, δ is the illumination weight, and l is the illumination sensitivity adjustment coefficient. When the target moves violently or the illumination condition is poor, the detection result is usually unreliable. The adaptive factor $c_{k}$ increases, the confidence decreases, and ASORT automatically increases the effective measurement noise covariance $\hat{R}$. That is, the weight of the current unreliable measurement value is reduced during state update, making the filtering result more dependent on the predicted value, thereby improving the adaptability and robustness of the algorithm in complex dynamic environments.

In the ASORT dynamic target tracking framework, the Hungarian algorithm serves as the core engine for multi-target association. It achieves accurate association between detection boxes and predicted trajectories through a global optimal matching strategy, with its specific process as follows:

First, two sets are determined. The first one is the detection box set:

(22) $$Z=\left\{Z_{1},Z_{2},\ldots ,Z_{i}\ldots ,Z_{m}\right\}$$

where $Z_{i}$ denotes the state vector of the i-th dynamic object bounding box in the current frame output by the target detection network.

In addition, the predicted trajectory set needs to be determined:

(23) $$Y=\left\{Y_{1},Y_{2},\ldots Y_{j}\ldots ,Y_{n}\right\}$$

where $Y_{j}$ denotes the state vector of the j-th predicted bounding box generated based on the tracking result of the previous frame and the AKF (Adaptive Kalman Filter). Then, the IOU (Intersection over Union) is used to measure the spatial overlap:

(24) $$IOU\left(Z_{i},Y_{j}\right)=\frac{A\left(Z_{i}\cap Y_{j}\right)}{A\left(Z_{i}\cup Y_{j}\right)}$$

The numerator is the intersection area of the two boxes, and the denominator is their union area. A larger value indicates a higher degree of spatial consistency.

To adapt to the goal of the Hungarian algorithm, which is to minimize the total cost, the matching cost function is defined as follows:

(25) $$C\left(Z_{i},Y_{j}\right)=1-IOU\left(Z_{i},Y_{j}\right)$$

A cost matrix C of dimension m is generated, with its element $C_{ij}=C\left(Z_{i},Y_{j}\right)$. Then, the optimal matching is solved on the cost matrix C, and the set of optimal matching pairs $\left\{\left(Z_{i},Y_{j}\right)\right\}$ is output.

The matching results are divided into three categories:

If the pairing is successful, for the successfully matched $\left(Z_{i},Y_{j}\right)$ pairs, the detection box $Z_{i}$ is used as a new observation value and input into the AKF corresponding to $Y_{j}$ to update the state vector and covariance matrix.

If there are unmatched detection boxes $Z_{i}$, they are regarded as new targets, and new trajectories are initialized with the initial state set based on $Z_{i}$.

If there are unmatched predicted trajectories $Y_{j}$, a loss counter is activated. If the number of consecutive lost frames exceeds the set threshold, the trajectory is deleted; otherwise, it is retained to wait for subsequent matching.

Finally, the set of all valid bounding boxes B and motion states in the current frame are output.

#### 2.2.2. Dynamic Feature Point Elimination

Based on the state of the bounding box, it is directly applied to the full set of feature points extracted by the front-end optimized SuperPoint, with the filtering rules as follows:

If the bounding box is stationary or no bounding box is detected, all feature points extracted by SuperPoint are retained. If a moving bounding box is detected, the set of feature points extracted by SuperPoint is traversed, and the points whose pixel coordinates are within the dynamic bounding box are eliminated.

**Figure 5:**
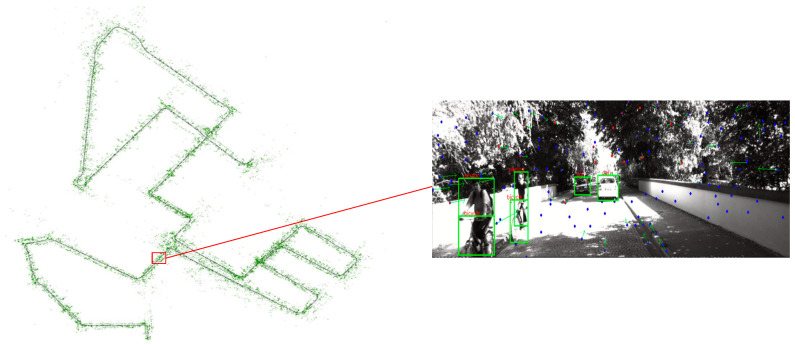
Comparative effect diagram of dynamic feature point rejection.

This filtering mechanism can efficiently remove dynamic interfering features. In the KITTI-08 sequence test, the dynamic feature point elimination rate reaches 92.3%, providing pure static background feature inputs for the back-end optimization of visual–inertial navigation and effectively reducing the positioning error in dynamic scenarios. The effect diagram is shown in Figure 5, where the green box on the right represents the detected dynamic object, and static feature points are retained after eliminating it during feature extraction.

## 3. Experiments

To evaluate the algorithm performance, experiments are conducted in this paper on the public KITTI dataset. The experiments mainly focus on localization accuracy, algorithm robustness, real-time performance, and comparisons with existing mainstream algorithms.

### 3.1. Experimental Setup

The public KITTI dataset was selected for experimental validation: Focusing on outdoor dynamic traffic scenarios, the KITTI dataset is equipped with an on-board stereo camera and an IMU. This paper focuses on testing 8 sequences (00, 02, 05, 06, 07, 08, 09, 10) that contain dynamic objects such as moving vehicles and pedestrians. The comparison algorithm adopted was VINS-Fusion, an optimization-based VIO algorithm. The evaluation metrics included ATE (Absolute Trajectory Error) and processing time. The experimental hardware platform consisted of an Intel Core i7-9700K CPU @ 3.60GHz and an NVIDIA GeForce RTX 3060 Ti GPU, running on the Ubuntu 18.04 operating system. This setup fully covers the requirements for verifying the algorithm’s performance, accuracy, and real-time capability in indoor and outdoor dynamic scenarios. Evaluation Metrics included the ATE and processing time.

ATE is a core metric for measuring the global absolute accuracy of localization algorithms, used to quantify the overall deviation between the estimated trajectory and the ground-truth trajectory. In the experiment, the estimated trajectory output by the algorithm and the ground-truth trajectory provided by the KITTI dataset were first obtained. A transformation matrix from the estimated pose to the reference pose was calculated using the least squares method to align the estimated positions with the reference positions. For the i-th timestamp in the navigation trajectory, let the 3D coordinates of the reference trajectory at this timestamp be $P_{ref,i}=(x_{ref,i},y_{ref,i},z_{ref,i})$, and the 3D coordinates of the trajectory to be evaluated be $P_{est,i}=(x_{est,i},y_{est,i},z_{est,i})$. The Euclidean distance between the estimated position and the reference position was calculated for each timestamp to obtain the APE (Absolute Position Error) at each moment:

(26) $$APE=\sqrt{{\left(x_{ref,i}-x_{est,i}\right)}^{2}+{\left(y_{ref,i}-y_{est,i}\right)}^{2}+{\left(z_{ref,i}-z_{est,i}\right)}^{2}}$$

Let there be a total of n timestamps. The RMSE (Root Mean Square Error) of the APE across all timestamps was computed to obtain the ATE:

(27) $$ATE=\sqrt{\frac{1}{n}\sum_{i=1}^{n}APE^{2}}$$

Ultimately, ATE reflects the cumulative error of the algorithm during long-term operation. It directly indicates the degree of consistency between the localization results and the real environment; even if local feature matching is disturbed by dynamic objects, ATE can reveal long-term stability defects of the system through global trajectory comparison.

### 3.2. Analysis of Localization Accuracy in Dynamic Scenarios

The performance of the proposed algorithm and the baseline algorithm VINS-Fusion in terms of localization accuracy on the KITTI dataset is presented in Table 1.

**Figure 6:**
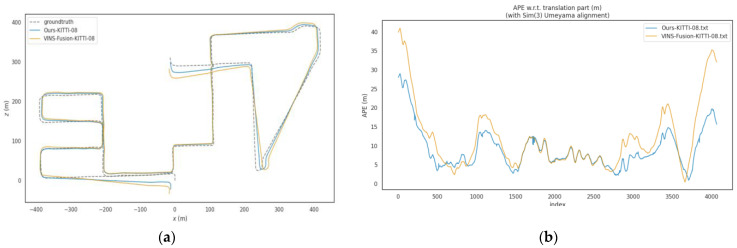
(**a**) Trajectory comparison figure of KITTI-08 sequence; (**b**) APE Comparison Figure of KITTI-08 Sequence.

**Figure 7:**
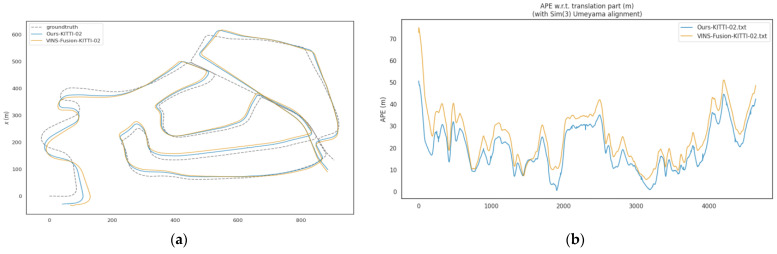
(**a**) Trajectory comparison figure of KITTI-02 sequence; (**b**) APE Comparison Figure of KITTI-02 Sequence.

In dynamic sequences of the KITTI dataset (including 00, 02, and 05 to 10), the ATE of the proposed algorithm is significantly lower than that of VINS-Fusion. The average ATE of VINS-Fusion in these sequences is approximately 12.44 m, while the average ATE of the proposed method is about 10.59 m, representing an average improvement of approximately 14.8% in localization accuracy. Among these sequences, the optimization effect is most prominent in the KITTI-08 sequence: the ATE decreases from 14.14 m to 10.55 m, with a localization accuracy improvement of about 25.4%, as shown in Figure 6. For the KITTI-02 sequence, the ATE reduces from 28.53 m to 22.15 m, achieving a localization accuracy improvement of approximately 22.4%, as illustrated in Figure 7. This result verifies the effectiveness of the dynamic point elimination mechanism and multi-scale feature optimization in suppressing dynamic interference and improving global localization accuracy. It should be noted that the degree of improvement varies across different sequences, which is closely related to the dynamic characteristics of each sequence—for example, some sequences have sparse dynamic objects with less mutual occlusion, while others feature dense dynamic objects and frequent interactions. Despite these differences, the proposed algorithm overall outperforms VINS-Fusion in all dynamic sequences, confirming its stable effectiveness in mitigating dynamic interference.

### 3.3. Validation of Module Effectiveness via Ablation Experiments

To explore the independent contributions and synergistic effects of the two core modules—the optimized SuperPoint feature extractor and the ASORT dynamic point elimination algorithm—ablation experiments were conducted on the representative sequences (00, 02, 08) of the KITTI dataset. Two experimental configurations were designed for comparison: the first one, denoted as SV, integrates the optimized SuperPoint while disabling the ASORT dynamic point elimination algorithm; the second one, labeled as YKV, incorporates the ASORT dynamic point elimination algorithm but disables the optimized SuperPoint, instead adopting the original feature point extraction algorithm. The experimental results are detailed in Table 2. By comparing the ATE performances of the SV configuration, YKV configuration, and the proposed algorithm in this paper, the independent effectiveness of each core module and the synergistic gain generated by their combination were analyzed in a quantitative manner.

The experimental results demonstrate that SV, YKV, and the proposed algorithm (which integrates these two modules) all significantly reduce the ATE in dynamic scenarios, with an overall performance gradient of “single module effective, synergy more optimal”. When SV operates independently, its ATE values on the three datasets are 16.52 m, 23.64 m, and 12.16 m, respectively, achieving an average improvement of approximately 13.8% in localization accuracy compared to the VINS-Fusion baseline. This validates that multi-scale feature optimization effectively enhances the robustness of static background features. When YKV operates independently, its ATE values are 16.24 m, 25.44 m, and 13.26 m, respectively, with an average localization accuracy improvement of about 9.6% over the baseline. This indicates that dynamic point elimination itself exerts a positive effect on localization accuracy; however, due to the limitations of the original feature extraction capability, its performance in complex scenarios is weaker than that of SV. The proposed algorithm, which combines the two modules, further amplifies the advantages: its ATE values are 15.57 m, 22.16 m, and 10.52 m, respectively, representing an average improvement of approximately 21.1% in localization accuracy compared to the VINS-Fusion baseline. Moreover, it achieves an additional 7.3% improvement compared to SV operating independently, and an extra 11.5% improvement compared to YKV operating independently.

### 3.4. Parameter Sensitivity Analysis

To verify the impact of the core parameters (β, δ, l) of the adaptive noise adjustment mechanism in the ASORT algorithm on system performance, a parameter sensitivity experiment was conducted based on three representative sequences (00, 02, 08) of the KITTI dataset. The ATE was adopted as the evaluation metric, and the control variable method was employed—fixing the other parameters at their optimal values while adjusting the target parameter individually. The parameter value ranges were determined by combining the conventional intervals in the field and the characteristics of the dataset: the motion weight factor β was set to [0.4, 0.8] (with a step size of 0.1), which covers the statistical distribution interval of the motion speeds of dynamic targets in the KITTI dataset, enabling full verification of the parameter adaptability under different motion intensities; the illumination weight factor δ was selected as [0.2, 0.6] (with a step size of 0.1), referring to the conventional weight allocation range for illumination interference evaluation in the field of computer vision while matching the sample proportion characteristics of different illumination scenarios in the KITTI dataset; the illumination sensitivity coefficient l was chosen as [1.0, 1.4] (with a step size of 0.1), determined according to the variation law of detection confidence when the illumination intensity deviates from the ideal value in the KITTI dataset, which can effectively verify the robustness of illumination response sensitivity. The results of the parameter sensitivity analysis are presented in Table 3.

The maximum performance fluctuation is calculated as (Maximum ATE when the parameter deviates from the optimal value−Optimal ATE)/Optimal ATE × 100%. As can be seen from Table 3, when the motion weight β is adjusted within [0.4, 0.8] with a step size of 0.1, the ATE values of sequences 00, 02, and 08 exhibit a trend of first decreasing and then slightly increasing. Specifically, KITTI-00 and KITTI-08 achieve the optimal ATE values of 16.24 m and 13.26 m, respectively, at β = 0.5, while KITTI-02 reaches the minimum ATE of 25.31 m at β = 0.6, with a maximum performance fluctuation of only 2.9%. This indicates that β primarily functions to balance the observation weights of high-speed and low-speed targets without exerting a decisive impact on the core performance of the algorithm. For the illumination weight δ adjusted within [0.2, 0.6] (step size 0.1), the optimal ATE values vary across sequences: KITTI-00 and KITTI-02 obtain the optimal ATE of 16.24 m and 25.44 m, respectively, at δ = 0.3, while KITTI-08 achieves the lowest ATE of 13.20 m at δ = 0.4, and the maximum fluctuation does not exceed 3.3%, suggesting that stable performance can be achieved by matching the distribution characteristics of illumination scenarios in the dataset. When the illumination sensitivity l is tuned within [1.0, 1.4] (step size 0.1), the optimal ATE values also show sequence-specific characteristics: KITTI-00 and KITTI-02 reach the optimal ATE of 16.24 m and 25.44 m, respectively, at l = 1.1, while KITTI-08 achieves the minimum ATE of 13.18 m at l = 1.2, with a maximum fluctuation of less than 2.6%, demonstrating that the adaptive adjustment logic can effectively mitigate illumination interference even with slight parameter deviations. Overall, when the core parameters fluctuate within reasonable ranges with a step size of 0.1, the maximum ATE fluctuation of the algorithm is only 3.3%, which is significantly lower than the 9.6% accuracy improvement achieved by the standalone ASORT algorithm in the ablation experiment (Section 3.3). This fully confirms that the core advantage of the ASORT algorithm originates from its adaptive adjustment logic rather than the precise tuning of individual parameters, and the algorithm possesses excellent robustness against parameter perturbations.

### 3.5. Real-Time Performance Analysis

Real-time performance is a critical requirement for the practical application of visual–inertial navigation systems. During the experiment, the average single-frame image processing time of the proposed method and the comparison algorithm VINS-Fusion when processing each dataset sequence was recorded, and the results are presented in Table 4.

It can be concluded from the table that the average single-frame processing time of the proposed algorithm in KITTI outdoor scenarios is approximately 23.9 ms, which meets the real-time performance requirements. Compared with the VINS-Fusion algorithm, the processing time of the proposed method increases by an average of about 9.3 ms. This increment mainly stems from two aspects: first, the improved SuperPoint feature extraction network is more computationally complex than the original front-end of VINS-Fusion; second, the ASORT dynamic point elimination module introduces additional computational load. In terms of the trade-off between efficiency and accuracy, although the computational time increases, the proposed method achieves a significant improvement in localization accuracy in dynamic scenarios, as shown in the previous accuracy analysis. Therefore, the increased time consumption can be regarded as a reasonable cost for obtaining higher accuracy and robustness, and future work will focus on optimizing the computational efficiency of these two modules.

## 4. Conclusions

To address the issue of reduced localization accuracy in VIO algorithms caused by moving object interference in dynamic scenarios, this paper proposes an improved algorithm integrating multi-scale feature optimization and real-time dynamic point elimination. The conclusions are as follows:

First, to address the limitation of insufficient discrimination between static and dynamic features in the original SuperPoint, we optimized the SuperPoint encoder by designing a dual-branch multi-scale feature fusion structure and integrating 1 × 1 convolutional channel compression. This optimization not only improves the quality of static background feature extraction but also refines the discrimination of dynamic–static boundary features, reducing the misjudgment of boundary features that are easily confused in dynamic scenarios. This improvement is reflected in the experimental results as a reduction in invalid feature points input to the backend, laying a foundation for more accurate pose estimation. This can be effectively verified by comparing the “SV configuration (integrating only the optimized SuperPoint while disabling the ASORT dynamic point elimination algorithm)” with the “VINS-Fusion baseline” in ablation experiments: In the KITTI-00, 02, and 08 sequences, the ATE values of the SV configuration are 16.52352 m, 23.63574 m, and 12.15743 m, respectively, achieving an average improvement of approximately 13.8% compared to the VINS-Fusion baseline. This validates the role of the encoder optimization in enhancing the quality of static features and improving localization accuracy.

Second, the proposed ASORT algorithm addresses the tracking drift issue of the traditional SORT algorithm in dynamic scenarios. By adaptively adjusting the observation noise covariance (incorporating target motion state and illumination conditions) and performing robust multi-target matching via the Hungarian algorithm, it achieves more reliable real-time detection and tracking of dynamic objects in each frame, thereby accurately filtering out feature points associated with dynamic objects. This module reduces the interference of dynamic features on pose estimation, which can be effectively demonstrated by comparing the “YKV configuration (integrating only the ASORT dynamic point elimination algorithm while disabling the optimized SuperPoint and adopting the original feature point extraction algorithm)” with the “VINS-Fusion baseline” in ablation experiments: In the KITTI-00, 02, and 08 sequences, the ATE values of the YKV configuration are 16.24376 m, 25.44375 m, and 13.25846 m, respectively, achieving an average improvement of approximately 9.6% compared to the VINS-Fusion baseline. This proves the effectiveness of ASORT in suppressing dynamic interference.

Third, the integration of the two modules forms a “feature optimization-dynamic purification” collaborative mechanism: the optimized SuperPoint encoder provides high-quality feature candidates, while the ASORT algorithm further purifies these candidates by removing dynamic features, ensuring that only reliable static features participate in subsequent feature matching and backend nonlinear optimization. This synergy directly translates to improved localization accuracy, which can be effectively verified through the experimental results in Section 3.2: In the dynamic sequences (00, 02, 05–10) of the KITTI dataset, the ATE of the fused algorithm is significantly lower than that of VINS-Fusion. The average ATE decreases from approximately 12.44 m (baseline) to 10.59 m, representing an average improvement of about 14.8%. Specifically, in the KITTI-08 sequence, the ATE decreases from 14.14232 m to 10.54754 m (an improvement of approximately 25.4%), and in the KITTI-02 sequence, the ATE decreases from 28.53246 m to 22.14632 m (an improvement of approximately 22.4%). Meanwhile, ablation experiments show that the average ATE improvement of the fused algorithm (21.1%) is significantly higher than the independent contributions of the SV configuration (13.8%) and the YKV configuration (9.6%), fully demonstrating the synergistic gain of the “feature optimization-dynamic purification” mechanism.

Although the “feature optimization-dynamic purification” collaborative framework proposed in this paper demonstrates effectiveness in dynamic VIO, it still has three core limitations: First, the computational complexity is relatively high. The multi-scale feature fusion in the improved SuperPoint encoder, along with the AKF and Hungarian matching mechanisms in the ASORT algorithm, introduce additional computational burdens, making real-time performance susceptible to constraints in resource-constrained scenarios. Second, there is strong module dependency. The algorithm’s performance relies on the accuracy of the target detection network, and the effect of dynamic feature elimination may be limited when deploying lightweight models on embedded devices. Third, the adaptability to dynamic scenes is limited. The robustness of ASORT against sudden extreme motions and complex occlusion scenarios still needs improvement.

To address these issues, future research will focus on three aspects: First, lightweight model design, which involves compressing the SuperPoint encoder and target detection network through neural architecture search, adaptive pruning, and dynamic inference mechanisms to balance accuracy and efficiency. Second, algorithm optimization, including constructing an end-to-end dynamic feature processing framework and developing cross-module knowledge distillation techniques to reduce dependence on a single module. Third, enhancing dynamic adaptability by introducing an attention-based motion prediction model, multimodal fusion strategies, and an adaptive computing framework to improve robustness in extreme scenarios and expand hardware adaptability. These directions are expected to overcome current limitations and promote the practical application of dynamic VIO technology in broader scenarios.

This study proposes an improved visual–inertial navigation algorithm based on the VINS-Fusion framework, integrating multi-scale feature optimization via a reconstructed SuperPoint encoder and real-time dynamic point elimination using the ASORT algorithm, which combines object detection, adaptive Kalman filtering, and Hungarian matching. Experiments on the KITTI dataset demonstrate improvement in absolute trajectory error, indicating enhanced accuracy and robustness in dynamic environments. While the work presents a solid technical contribution with thorough experimental validation, several issues need addressing to elevate its scholarly impact.

## Figures and Tables

**Table 1:** ATE Comparison on the KITTI Dataset.

Dataset	VINS-Fusion(m)	Ours(m)
KITTI-00	18.41124	15.03534
KITTI-02	28.53246	22.14632
KITTI-05	12.44625	12.14674
KITTI-06	12.73677	12.43485
KITTI-07	6.59655	6.12671
KITTI-08	14.14232	10.54754
KITTI-09	10.73765	9.86424
KITTI-10	7.75347	6.27634

**Table 2:** Ablation Study on ATE Comparison.

Dataset	VINS-Fusion(m)	SV(m)	YKV(m)	Ours(m)
KITTI-00	18.41124	16.52352	16.24376	15.56553
KITTI-02	28.53246	23.63574	25.44375	22.16476
KITTI-08	14.14232	12.15743	13.25846	10.52354

**Table 3:** Results of Parameter Sensitivity Analysis.

Parameters	Fixed Parameters (Global Optimal)	Parameter Values	KITTI-00 ATE (m)	KITTI-02 ATE (m)	KITTI-08 ATE (m)	Maximum Performance Fluctuation
Motion weight (β)	δ = 0.3, l = 1.1	0.4	16.61	26.03	13.52	2.9%
		0.5	16.24	25.44	13.26	
		0.6	16.32	25.31	13.34	
		0.7	16.41	25.75	13.46	
		0.8	16.55	25.92	13.61	
Illumination weight (δ)	β = 0.5, l = 1.1	0.2	16.59	26.09	13.50	3.3%
		0.3	16.24	25.44	13.26	
		0.4	16.36	25.58	13.20	
		0.5	16.48	25.74	13.49	
		0.6	16.63	25.91	13.64	
Illumination sensitivity (l)	β = 0.5, δ = 0.3	1.0	16.55	26.05	13.47	2.6%
		1.1	16.24	25.44	13.26	
		1.2	16.33	25.56	13.18	
		1.3	16.45	25.71	13.47	
		1.4	16.59	25.88	13.52	

**Table 4:** Comparison of Average Single-Frame Computation Time.

Dataset	VINS-Fusion(m)	Ours(m)
KITTI-00	14.865	24.208
KITTI-02	14.697	23.556
KITTI-05	14.535	24.135
KITTI-08	14.745	23.286
KITTI-10	14.475	24.645

## Data Availability

Data are contained within the article.

## Abbreviations

The following abbreviations are used in this manuscript:

VIO	Visual–Inertial Odometry
IMU	Inertial Measurement Unit
SORT	Simple Online and Realtime Tracking
ASORT	Adaptive Simple Online and Realtime Tracking
RGB	Red, Green, Blue
VGG	Visual Geometry Group
IOU	Intersection over Union
ATE	Absolute Trajectory Error
APE	Absolute Position Error
RMSE	Root Mean Square Error
